# Frequency and change mechanisms of psychotherapy among depressed patients: study protocol for a multicenter randomized trial comparing twice-weekly versus once-weekly sessions of CBT and IPT

**DOI:** 10.1186/s12888-015-0532-8

**Published:** 2015-06-30

**Authors:** Sanne J. E. Bruijniks, Judith Bosmans, Frenk P. M. L. Peeters, Steven D. Hollon, Patricia van Oppen, Michael van den Boogaard, Pieter Dingemanse, Pim Cuijpers, Arnoud Arntz, Gerdien Franx, Marcus J. H. Huibers

**Affiliations:** 1 Department of Clinical Psychology, VU University Amsterdam, and EMGO Institute, Amsterdam, The Netherlands; 2 Department of Health Sciences, EMGO Institute for Health and Care Research, Faculty of Earth and Life Sciences, Section of Health Economics and Health Technology Assessment, VU University Amsterdam, Amsterdam, The Netherlands; 3 Department of Psychiatry and Psychology, University Hospital Maastricht; School for Mental Health and Neuroscience, Faculty of Health, Medicine and Life Sciences, Maastricht University, Maastricht, Netherlands; 4 Department of Psychology, Vanderbilt University, Nashville, Tennessee USA; 5 Department of Psychiatry, VU Medical Centre/GGZ ingeest, and the EMGO Institute, Amsterdam, The Netherlands; 6 PsyQ, Parnassia Groep, Hague, Netherlands; 7 Altrecht Institute for Mental Health Care, Utrecht, Netherlands; 8 Department of Clinical Psychology, University of Amsterdam, Amsterdam, The Netherlands; 9 Trimbos-institute, Netherlands Institute of Mental Health and Addiction, Utrecht, The Netherlands

**Keywords:** Depression, Intensity, Change mechanisms, Psychotherapy, Physiology, Multicenter randomized controlled trial

## Abstract

**Background:**

Cognitive behavioral therapy (CBT) and interpersonal therapy (IPT) are among the most well established therapies for the treatment of depression. However, some major questions remain unanswered. First, it is unknown what session frequency results in the most optimal (cost) effectiveness in psychotherapy. Second, the debate as to what mechanisms underlie the effect of psychotherapy has not yet been resolved. Enhancing knowledge about the optimal session frequency and mechanisms of change seems crucial in order to optimize the (cost) effectiveness of psychotherapy for depression. This study aims to compare treatment outcome of twice-weekly versus once-weekly sessions of CBT and IPT. We expect twice-weekly sessions to be more effective and lead to more rapid recovery of depressive symptoms in comparison to once-weekly sessions. Both therapy-specific and non-specific process measures will be included to unravel the mechanisms of change in psychotherapy for depression. Besides the use of self-reports and behavioral observations, this study will also examine underlying biological processes by collecting blood samples.

**Method:**

In a multicenter randomized trial, two hundred depressed patients will be recruited from Dutch specialized mental healthcare centers and randomized into one of the following groups, all receiving a maximum of 20 sessions in different frequencies: a) twice-weekly sessions at the start of CBT, b) twice-weekly sessions at the start of IPT, c) once-weekly sessions at the start of CBT, d) once-weekly sessions at the start of IPT. Primary outcome measures are depression severity, cost-effectiveness and quality of life. Process measures include therapeutic alliance, recall, therapy-specific skills, motivation and compliance. Assessments will take place during baseline, monthly during treatment and follow-up at month 9, 12 and 24. In addition, at 12 and 24 months, the frequency of depressive episodes in the previous year will be assessed. Blood samples will be taken pre- and post-treatment. The study has been ethically approved and registered.

**Discussion:**

Finding that twice-weekly sessions are more effective or lead to more rapid recovery of depressive symptoms could lead to treatment adaptations that have the potential to reduce the personal and societal burden of depression. In addition, insight into the mechanisms of change and physiological processes in psychotherapy will enable us to optimize treatments and may help to understand human functioning beyond the context of treatment.

**Trial registration:**

The study has been registered on October 21th, 2014 at the Netherlands Trial Register, part of the Dutch Cochrane Centre (NTR4856).

## Background

Cognitive (behavioral) therapy (CBT) [1] and interpersonal therapy (IPT) [2] are among the most well established short-term therapies for the treatment of depression. Both treatments are effective and are recommended as first-choice treatments [3–6]. However, despite the efficacy and the acceptance of these therapies among researchers and clinicians, some major questions remain unanswered. This study will address two questions about the effectiveness and change mechanisms of psychotherapy for depression. First, there is a lack of clarity about the optimal intensity of psychotherapy [7]. It seems important to know what session frequency will provide the most optimal (cost) effectiveness in psychotherapy as this could lead to more rapid recovery and a reduction of direct (e.g. health care) and indirect costs (e.g. fewer work-days lost). Second, there are still many uncertainties regarding how the psychotherapies actually work. Despite the numerous efforts to understand the change mechanisms in psychotherapy, this topic remains largely unresolved [8–13]. It is not clear if and to what extent therapy- specific elements (e.g. cognitive skills in CBT or relational skills in IPT), non-specific elements (e.g. therapeutic alliance), or both, contribute to the effects of psychotherapy. Moreover, there is an ongoing discussion about what methods are necessary to conduct a successful study on the mechanisms of change in psychotherapy [14]. However, when investigating mechanisms of change during psychotherapy it seems important to not only include patient self-report measures, but to investigate multiple layers of behavior (e.g. independent raters, biological measurements) [15]. This study aims to compare treatment outcomes of twice-weekly versus once-weekly sessions of two widely used types of psychotherapy: CBT and IPT. Both therapy- specific and non-specific process measures will be included to enhance clarity about the mechanisms of change in psychotherapy for depression. In addition, we will collect blood samples in order to investigate the (predictive) role of several physiological processes (e.g. oxytocin, brain-derived neurotrophic factor, DNA methylation and RNA markers) during the course of psychotherapy for depression. The main research questions are:

- What is the (cost) effectiveness of twice-weekly sessions versus once-weekly sessions at the start of psychotherapy for major depression (CBT or IPT) over the course of 24 months?
- What change mechanisms may be involved in mediating the effects of psychotherapy?

### Frequency of psychotherapy

Several studies have shown an association between the frequency of therapy sessions and symptom improvement [16–18]. More specifically, a recent meta-regression found a strong and positive association between the number of sessions per week and the effect size of psychotherapy for adult depression. In that study it was session frequency, in contrast to the total number of sessions, which was related to treatment outcome [7]. In routine mental health care, therapy sessions are commonly planned once a week. The original CBT manual by Beck does recommend two sessions a week in the beginning of therapy [1]. Most empirical trials (mostly from the US) have established that CBT is efficacious providing twice-weekly treatment [19, 20]. However, Beck’s guidelines have not been followed consistently and Dutch mental health guidelines do not even give an indication of the frequency of psychotherapy for depression [6]. Given this gap between recommended and actual practice, it seems important to investigate whether a higher intensity of treatment will lead to better and more rapid treatment outcomes and is related to lower rates of relapse. The question is whether the results of the controlled clinical trials can generalize to clinical practice when an empirically-supported treatment is not implemented in the manner in which it was done. Although both conditions will have the same maximum number of sessions, it is conceivable that patients in the twice-weekly condition will respond more quickly and need fewer sessions than patients in the once-weekly condition. This could lead to a reduction of both direct (e.g. health care) and indirect costs (e.g. fewer work-days lost). In addition, differences in treatment intensity might not only lead to differences in the length of treatment, number of sessions and a reduction of costs but also to the activation of different components of therapy within different phases of the treatment. It is possible that some components of therapy will become more effective or present when patients receive sessions twice a week instead of once a week. Furthermore, it will be interesting to see whether therapy type (CBT vs IPT) moderates the effect of initial session frequency (twice-weekly versus once-weekly). This study will be the first in the field of depression that directly tests the hypothesis that twice-weekly sessions lead to better outcomes than once-weekly sessions. If we find twice-weekly sessions to be more effective or to lead to more rapid recovery, this could lead to treatment adaptations that have the potential to reduce the societal burden of depression. In addition, the question why a particular session frequency is more effective than the other might lead to new directions for understanding change mechanisms in psychotherapy.

### Mechanisms of change

Nowadays, a broad range of therapies is available for the treatment of depression. Research points to the equal effectiveness of different psychotherapies (on average) and the number of effective psychotherapies seems to be even expanding [3, 21]. The fact that different psychotherapies stemming from different theoretical backgrounds seem to produce comparable outcomes (on average) raises the question as to whether the different treatments might work through common non-specific factors (e.g. therapeutic alliance) instead of the therapy-specific techniques (e.g. change in cognitive skills for CBT or relational skills for IPT) that are assumed to underlie the effects of a specific psychotherapies. However, after thirty years of meta- analyses that tried to support the notion that treatment efficacy is due to non-specific factors, the controversy remains [9, 12]. Critics point to the limitations of a design that includes pre- and post- assessments only, as if often the case in randomized controlled trials. Several methodological recommendations have been made to improve the quality research on change mechanisms in psychotherapy. First, mediators have to be monitored across the course of treatment and not only before and after [10]. The timing of these assessments is critical: change in the mediator must occur before change in the outcome in order to rule out reverse causality. To ensure that the change observed in the mediator was not the result of a causal path from early change in the outcome variable to subsequent change in the mediator, the measurement of the outcome measures should parallel the assessments of the change mechanisms [11, 13, 22]. Second, the inclusion of moderators into the research design could lead to new hypotheses about potential mediators [13, 22]. Third, studies should make use of more advanced statistical techniques that are capable of detecting causal mediation. An example includes growth mixture modeling [10]. This study will include both potential therapy-specific and non-specific mechanisms of change. These mechanisms will be measured after the first two weeks of treatment and once a month along with the outcome measures. We will include moderators that are expected to influence the direction or strength of the relation between the intervention and outcome. Furthermore, in order to analyze the mechanisms of change we will use a combination of structural equation modeling (SEM) and multilevel modeling [23]. In this way, we hope to provide the optimal conditions for detecting mechanisms of change in psychotherapy for depression within a multicenter randomized trial.

**Fig. 1:**
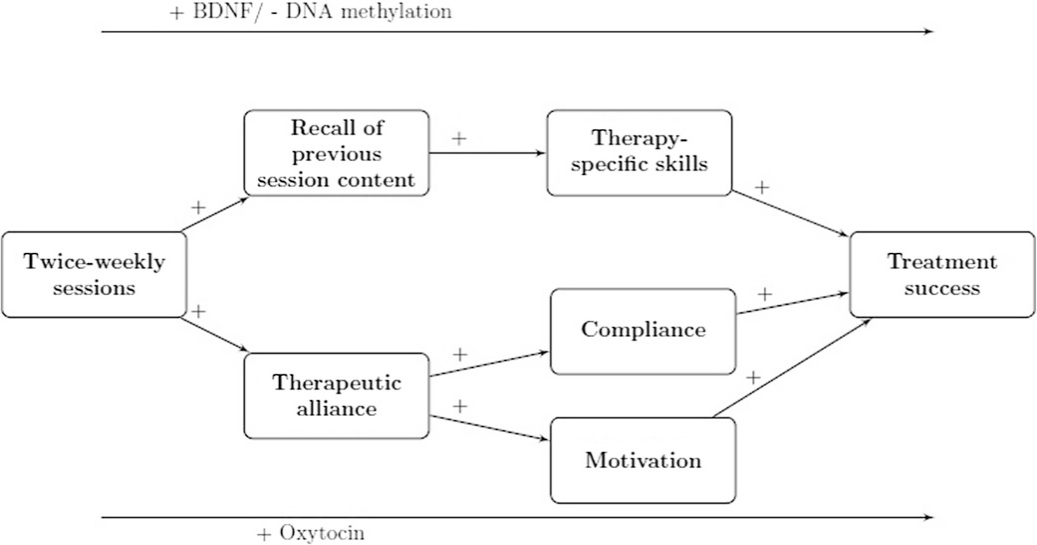
Hypothesized pathways of change in psychotherapy for depression

In this study, we hypothesize two pathways of change to be involved in the course of psychotherapy for depression (Fig. 1). First, we expect that a higher session frequency will enhance the quality of the relationship between patient and therapist. This relationship has been referred to as therapeutic alliance and involves the behaviors and processes within the session, patients’ and therapists’ objectives about therapy and the interpersonal attachment of mutual trust, confidence and acceptance. Previously, therapeutic alliance has been positively related to treatment outcome [24–26] while lower therapeutic alliance was associated with dropout [27]. Possibly, the relationship between patient and therapist will develop more rapidly when the contact is more intense. We expect that a better therapeutic alliance will lead to more patient motivation and compliance, as the therapist will have better opportunities to detect patient’s struggles in motivation and compliance. Subsequently, higher rates of motivation and compliance are expected to lead to better and faster treatment outcomes. The second pathway of change focuses on the process of learning. It is possible that a higher session frequency will increase patients’ learning processes resulting in better treatment outcomes. This seems congruent with previous studies that show that it is the survival of neurons born in the last five days that is important for learning [28]. Possibly, a higher frequency will increase the survival of neurons that are important for learning and as a consequence optimize learning processes. In this study, we will measure the degree the patient is able to recall the content of the previous session and the therapeutic-specific skills of the patient (e.g. cognitive therapy skills or interpersonal therapy skills). We expect that a higher session frequency will lead to better session recall that, as a consequence, will lead to the development of better therapy- specific skills. Better therapy-specific skills are expected to lead to better and faster treatment outcomes.

### Physiological pathways in the course of psychotherapy for depression

In addition to the focus on self-reports or behavioral observations for detecting mechanisms of change, this study also aims to focus on deeper layers of behavior. Earlier studies showed that a variety of physiological levels are deregulated in depressed patients and can be restored with use of antidepressants [29–36]. In this study we will investigate the role of the brain-derived neurotrophic factor (BDNF) [30–32], methylation profile of the BDNF gene (e.g. CpG I and IV) [33, 34] and oxytocin [35, 36] and RNA marker profiles [37] during psychotherapy for depression. We expect levels of BDNF and oxytocin to restore during treatment (potential mediators) while the methylation profile of the BDNF gene (e.g. CpG I and IV) and RNA marker profiles might play a role in the prediction of treatment outcome (prognostic indices) and quite possibly differential treatment outcome (potential moderators).

## Method

### Design of the study

**Fig. 2:**
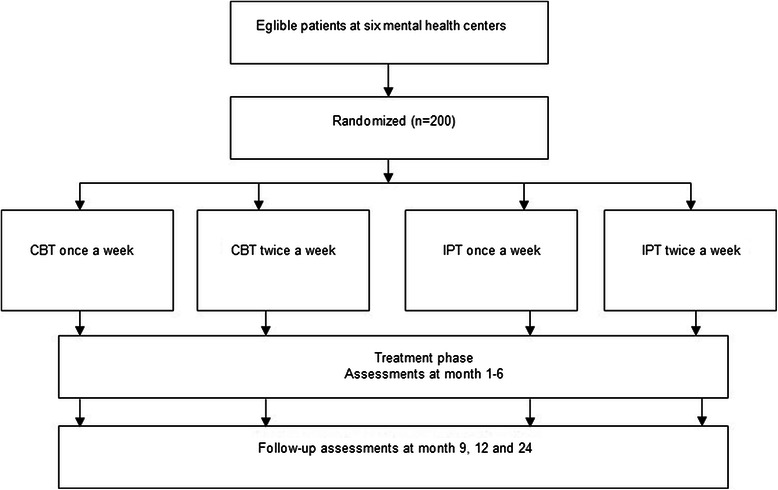
Flow of participants

We will conduct a multicenter randomized trial with four parallel groups: a) twice-weekly sessions at the start of CBT (*n* = 50), b) twice-weekly sessions at the start of IPT (*n* = 50), c) once-weekly sessions at the start of CBT (*n* = 50), d) once-weekly sessions at the start of IPT (*n* = 50). The anticipated flow of subject enrolment is graphically shown in Fig. 2. In essence, this is a 2 × 2 factorial design that allows the comparison of frequency type (i.e. twice-weekly vs once-weekly) in the combined psychotherapy groups (i.e. CBT and IPT) and vice versa. The Medical Ethics Committee of VU University Amsterdam approved the study protocol (registration number 2014.337). The study is registered at the Netherlands Trial Register, part of the Dutch Cochrane Centre (NTR4856).

### Participants

We aim to include 200 patients that satisfy the following criteria: a. DSM-IV or DSM-V diagnosis of a major depressive disorder (MDD) (including chronic depression) or DSM-V diagnosis of a persistent depressive disorder, b. age > 18 c. the patient is currently not using antidepressants or the use of antidepressants has been unchanged for at least three months before start of treatment and no change in the use of antidepressants is planned, d. sufficient knowledge of the Dutch language, f. score > 19 on Beck’s Depression Inventory II (BDI-II) [37–39]. Patients will be excluded when a. there is a high risk of suicide according to the intake staff, b. they are diagnosed with drug or alcohol dependence, c. they have a primary diagnosis other than MDD, d. they are diagnosed (or suspected) with a cluster A or B personality disorder during the intake staff, e. they had psychotherapy (CBT or IPT) focusing on a major depressive disorder in the previous year or f. they have no access to internet facilities. The presence of a depressive disorder will be indicated by a diagnosis following the Structural Clinical Interview for DSM-IV Axis I disorders (SCID-I) [40] or Mini International Neuropsychiatric Interview Plus (MINI-Plus) [41].

### Sample size

Based on the meta-analysis of treatment intensity [7], we estimate the post-treatment effect size for the difference in depressive symptoms (BDI-II) between the main conditions (twice- versus once-weekly in the combined psychotherapy groups) to be 0.45. Based on this effect size (alpha = 0.05, power (1-ß) = 0.80, two-tailed), we need 79 participants per group (e.g. once versus twice-weekly sessions). Taking 25 % dropout into account, this means in total at least 200 depressed patients. The sample size should also suffice to detect possible interactions of a comparable effect size between treatment conditions.

### Recruitment

Patients will be recruited in the Dutch specialized mental healthcare centers including GGZ inGeest (Amsterdam), Riagg Maastricht (Maastricht), Altrecht (Utrecht) and PsyQ (Haarlem, The Hague, Leiden). During the intake, patients will be checked for the in- and exclusion criteria. If patients are eligible they will be approached for participation in the study and receive a general information folder. Patients that cannot participate in the study treatment will receive treatment as usual (Fig. 2).

### Randomization and procedure

An independent research assistant will contact eligible patients one week after the intake procedure in order to give patients additional study information and to check if the patient is motivated to participate in the study. If no structured diagnostic interview has been completed yet during the intake procedure, the research assistant will plan a diagnostic interview (SCID-I/MINI). Subsequently, if the patient wants to participate and fulfills the inclusion criteria, the patient will be invited for an online baseline assessment and to sign informed consent. After the baseline assessment, the patient will be randomized in one of the four groups by using a computer script performing block randomization (e.g. one- versus twice weekly; CBT versus IPT). Randomization (patient level) will be pre-stratified according to severity (high severity = BDI score = > 30; low severity = BDI score < =29) and treatment site. A venipuncture by experienced nurses will be planned before the start of treatment. Besides the baseline assessment, the patient will be asked to complete monthly online assessments in the course of treatment (month 1–6) and follow-up assessments at month 9, 12 and 24. This means that the moments of assessment will be equal for each group. In addition, the therapist and patient will be asked to fulfill some questionnaires during the sessions. A second venipuncture will be planned after the end of treatment (month 6).

### Interventions

In the Netherlands, cognitive behavioral therapy (CBT) and interpersonal therapy (IPT) are the most frequently practiced evidence- based psychological interventions for depression. Both therapies are applied in weekly sessions in routine clinical settings. CBT for depression is based on the manual by Beck [1] and IPT is based on the manual by Klerman [2]. The interventions will be offered in the participating mental health centers. Participants in the experimental condition (twice-weekly sessions) will receive 16 sessions during the first 8 weeks of treatment, and 4 sessions during the last 8 weeks (up to 20 sessions during 16 weeks). Patients in the control condition (once-weekly sessions) will receive 16 sessions during the first 16 weeks and 4 sessions during the last 8 weeks (up to 20 sessions during 24 weeks). Treatment manuals are used for all interventions and both treatments will contain 12 to 20 sessions of 45 minutes, depending on the individual progress of the patients. Qualified therapists who received additional training from prof. Steven Hollon (CBT) and prof. Holly Swartz (IPT), experts in their respective modalities, will deliver the interventions. Therapists participate in only one of the therapy conditions (CT or IPT) and will consult each other on current cases in biweekly consultation meetings. The sessions will be videotaped.

### Instruments

An overview of all patient instruments per assessment is given in Table 1. An overview of therapist/observer ratings per assessment is given in Table 2.

**Table 1:** Overview of patient instruments per time point (months)

Instruments		0	5	1	2	3	4	5	6	9	12	24
*Clinical outcomes*												
*Depression*												
Beck Depression Inventory II (BDI-II)^1^		x	x	x	x	x	x	x	x	x	x	x
Remission of Depression Questionnaire (RDQ)		x				x			x	x	x	x
*Quality of life*												
RAND-36		x	x			x			x	x	x	x
EQ-5D(5L)		x				x			x	x	x	x
Happiness question^a^		x	x	x	x	x	x	x	x	x	x	x
Mood question^a^	x	x	x	x	x	x	x	x	x	x	x	
Longitudinal Interval Follow-up Evaluation (LIFE)		x	x	x	x	x	x	x	x	x	x	x
*Cost-Effectiveness*												
Trimbos/iMTA questionnaire for Costs associated with Psychiatric Illness - update version 2012 (TiC-P)		x				x			x	x	x	x
*Process/Predictors*												
*-Therapy-specific-Therapist-patient relationship*												
Working Alliance Inventory (WAI)			x	x	x	x	x	x	x			
Motivation												
Autonomous and Controlled Motivation for Treatment		x	x	x	x	x	x	x	x			
Questionnaire (ACMTQ)												
*Compliance*												
Patient compliance ratings			x	x	x	x	x	x	x	x		
*Behavioral activation*												
Behavioral Activation for Depression Scale (BADS)		x	x	x	x	x	x	x	x			
*Automatic thoughts*												
Cognition Checklist (CCL)		x	x	x	x	x	x	x	x			
*Emotion regulation*												
Action Control Scale (ACS)		x							x			
*Childhood trauma*												
Childhood Trauma Questionnaire (CTQ)		x										
Treatment preferences		x										
Treatment expectations		x										
*Learning-*												
*CT Skills*												
Competencies of Cognitive Therapy Scale (CCTS)		x	x			x			x			
*IPT Skills*												
IPT Skill Inventory		x	x			x			x			
*Executive functioning*												
N-back task		x	x	x					x			
*Biological markers*		x							x			
Brain-Derived Neurotrophic Factor (BDNF)												
DNA methylation		x							x			
Oxytocin		x							x			
*Other measures*												
*Demographics*		x										
*Diagnostics*												
Structured Clinical Interview for DSM-IV		x										
Axis I Disorder (SCID-I)/MINI-Plus												
*Attribution of therapy effects*									x			
*Treatment evaluation*									x			

^a^Depressive symptoms, mood and happiness will be rated each session

**Table 2:** Overview of therapist/observer instruments per time point (months)

Instruments	0	1	2	3	4	5	6	9	12	24
*Process/Predictors*										
*-Therapy-specific-*										
*Therapist-patient relationship*										
Working Alliance Inventory- Therapist (WAI-T)		x	x	x	x	x	x			
Working Alliance Inventory- Observer (WAI-O)		x	x	x	x	x	x			
*Compliance*										
Kept appointments										x
Replaced appointments										x
Therapist-rated compliance										x
*Therapist adherence*										
Collaborative Study Psychotherapy Rating Scale (CSPRS)										x
*-Learning-*										
*CT Skills*										
Competencies of Cognitive Therapy Scale-Therapist (CCTS-T)		x		x			x			
Performance of Cognitive Therapy Strategies (PCTS)										x
*Recall*										
Therapist-rated recall quality^a^		x	x	x	x	x	x			

Quality of recall will be assessed each session^a^

### Instruments: clinical outcome measures

#### Depression: Beck Depression Inventory II (BDI-II) [37–39, 42]

The BDI is a 21-item self-report instrument assessing depressive symptoms during the last two weeks. The items are rated from 0 to 3, with 3 for the most depressed mood. A score 0–13 indicates minimal depression, 14–19 mild depression, 20–28 moderate depression and 29–63 severe depression. The instrument can be answered in 5–10 minutes. Several studies have shown that the BDI-II also is a strong screening measure for depression [37, 42].

#### Quality of life: EQ-5D(5 L) [43, 44]

The EQ-5D consists of five health state questions (mood, symptoms of pain/discomfort, mobility, daily activities, self-care) on which respondents indicate their own health state on a scale from 0 (no problems) to 3 (severe problems) during the past week. In addition, patients will rate their overall health on a scale from 0 (worst possible health) to 100 (best possible health). The Dutch EQ-5D (5 L) tariff will be used to calculate Quality-Adjusted Life-Years (QALYs). The EQ-5D (5 L) was validated for the Dutch population [44].

#### Remission of depression: remission of depression questionnaire [45, 46]

The RDQ is a 41-item self-report questionnaire assessing symptoms of depression as well as other variables reported by patients as relevant to determining remission during the past week. The items are grouped into 7 domains: symptoms of depression (13 items), anxiety and irritability (5 items), features of positive mental health (11 items), coping ability (3 items), functioning (3 items), life satisfaction (3 items), and a general sense of wellbeing (3 items). Items are rated on a 3-point rating scale (not true (0) - always true (2)). The questionnaire has shown excellent psychometric qualities.

#### Quality of life: happiness question [47]

The question ‘If you were to consider your life in general, how happy or unhappy would you say you are, on the whole?’ can be rated on a 7-points scale (1 = completely unhappy – 7 = completely happy) and was used in 33 nation surveys, including the Netherlands. The happiness question (code = Code: O- HL/g/sq/v/7/a) was used in many studies. In addition, we will ask a happiness question focusing on the present day (‘How happy of unhappy would you say you are today?’).

#### Rand-36 [48]

The RAND-36 is an 36-item assessment of general health and disabilities and covers the following domains: psychical and social functioning, role restriction due to physical or emotional problems, mental health, energy, pain, and general health perception. The RAND-36 has shown to be a reliable, valid and sensitive measure [48].

#### Mood: mood question

Each session, patients will be asked to rate their current mood on a 0 (worst mood) -10 scale (best mood).

#### Longitudinal Interview Follow-up Evaluation (LIFE) [49]

The LIFE is a semi-structured interview that will be used by an independent rater to assess episodes and symptoms of depression retrospectively in the long-term follow-up phase. The LIFE has shown reliable and valid for characterizing the course of depression over the period of one year [50, 51].

### Instruments: cost-effectiveness

#### Trimbos/iMTA questionnaire for Costs associated with Psychiatric Illness (TiC-P) – updated version 2012 [52]

Societal costs in the past three months will be measured using the TiC-P. The TiC-P consists of two different parts: part one (16 items) compromises costs due to loss of production including absenteeism from paid and unpaid work, and presenteeism. Part two (4 items) focuses on use of (psychiatric) health care including primary and secondary care, complementary care and home care. For the valuation of health care utilization standard prices [53] will be used. Medication use will be valued using prices of the Royal Dutch Society for Pharmacy [54].

### Instruments: moderators and mechanisms of change

#### Therapist-patient relationship: working alliance inventory-short form [55–58]

The WAI intends to measure tasks (e.g. behaviors and cognitions that form the therapeutic process), bonds (e.g. positive personal attachments between patient and therapist) and goals (e.g. therapist and patient mutually endorsing and valuing the goals) as components of the therapeutic alliance. The questionnaire consists of 12 items rated on a 5-point Likert scale and will be filled out by both patient and therapist [59, 60]. All therapy sessions will be videotaped. Independent experts will rate the therapeutic alliance on a random selection of videotapes using the 12-item Observer version of the Working Alliance Inventory-Short (WAI-O-S). The instrument has shown to have adequate psychometric properties.

#### Motivation: autonomous and controlled motivation for treatment questionnaire [61]

The ACMTQ includes two six-item subscale in order to assess autonomous (identified; ‘I personally believe it is the most important aspect of becoming well’ and integrated; ‘Managing my depression allows me to participate in other important aspects of my life’) motivation and controlled (external; ‘Others would be upset if I didn’t” and introjective; ‘I would feel guilty if I didn’t do what my therapist said’) motivation.

The format of the questionnaire was adapted from the Treatment Self-Regulation Questionnaire (TRSQ; 61]. Patients are provided with a stem (‘I participate in therapy because’) and asked to rate twelve items on a 7-point rating scale (1 = strongly disagree - 7 = strongly agree). Internal consistency has been shown to be sufficient [62].

#### Compliance

Compliance will be operationalized as the amount of no-shows (not showing up without leaving a message), cancelled and replaced appointments. Patients will rate their effort in treatment before each session. Furthermore, the therapist will give a rating of patients compliance to the therapy at the end of treatment on a 7-point scale.

#### Activation: behavioral activation for depression scale [63]

The BADS is a 25-item self-report scale that intends to measure patients’ avoidance behavior and activity level during the past week. The scale includes four subscales: Activation, Avoidance/Rumination, Work- School and Social Impairment. Items are rated on a 7-point scale. Validity and reliability showed to be adequate [63].

#### Therapist adherence: collaborative study psychotherapy rating scale [64]

In order to measure therapists adherence to the treatment condition, independent raters will review a random selection of sessions by rating 76 items on a seven-point scale from the following subscales: Cognitive Behavioral Therapy (e.g. cognitive rationale, assessing cognitive processes, evaluating and changing beliefs, behavioral focus, homework and collaborative structure alternative cognitive strategies and operant approaches), Interpersonal Therapy (e.g. interpersonal rationale, focus on feelings, assessing interpersonal relationships and tendencies, assisting changes in interpersonal functioning, role transitions, interpersonal disputes, interpersonal deficits and interpersonal therapy scale ), Facilitative Conditions and Explicit Directiveness. Items regarding medication use and clinical management were omitted. High internal consistency and inter-rater reliability was reported [64].

#### Emotion Regulation: Action Control Scale 24 (ACS-24) [65, 66]

The ACS is a forced-choice self-report measure developed to assess differences in action-state orientation (the ability to initiate and maintain intentions). Two scales of the ACS will be used: action orientation subsequent to failure vs. preoccupation (AOF, 12 items, e.g. the ability to detach from thoughts about alternative goals or undesirable events that may interfere with progress on the task at hand.) and prospective and decision-related action orientation vs. hesitation (AOD, 12 items, e.g. the degree to which individuals have difficulty initiating intended goal-directed activities).

#### CT Skills: Competencies of Cognitive Therapy Scale-Self Report (CCTS-SR) [67, 68]

Two versions of this report will be used: a 29-item patient version and a 9-item therapist version. Both measures were designed to assess patients’ mastery of CT skills in the past two weeks. Patients rate the scale based on their skill use on a 1 (not at all) to 7 (completely) scale, while therapists evaluate a patient’s ability, independence, and frequency of use of behavioral activation, automatic thoughts, and core belief related CT strategies on a scale from 0 (none) to 6 (extensive). Psychometric properties seem promising [67].

#### Performance of CT Strategies (PCTS) [67]

Independent raters will assess the extent to which patients perform or intend to perform CT skills for a random selection of tapped sessions. The scale consist of 15 items that are rated on a 6-point Likert scale focusing on behavioral activation, automatic thought work, and schema or core belief work. Internal consistence has been shown to be sufficient [67].

#### IPT skill inventory

In order to explore the interpersonal skills the patients develops during treatment the IPT skill Inventory was developed by two authors (SB and FP) of the present paper. The questionnaire consists of 31 items that are rated on a 7-point Likert scale (not at all – completely). Items were constructed by use of the following subscales: general interpersonal skills (13 items), bereavement (4 items), interpersonal conflict (5 items), interpersonal change (5 items), and interpersonal deficiency (4 items). After data collection, a factor analysis will be conducted in order to investigate the structure of the questionnaire.

#### Recall

Each session, therapists will rate the degree to which the patient seems to remember last session’s content on 10-point scale (0 = patient remembers nothing – 10 = patient remembers everything).

#### Maltreatment in childhood: Childhood Trauma Questionnaire-Short Form (CTQ-SF) [69]

To assess maltreatment in childhood, the Childhood Trauma Questionnaire-Short Form (CTQ-SF) is used. The CTQ-SF is a 28-item retrospective self-report questionnaire designed to assess five types of negative childhood experiences: (1) emotional neglect, (2) emotional abuse, (3) physical neglect, (4) physical abuse and (5) sexual abuse. In addition, tendencies to minimize or deny abuse experiences are measured. The truth of each statement is rated on a 5-point scale. Adequate reliability and validity has been shown [70, 71].

#### Automatic Thoughts: Cognition Checklist (CCL) [72]

The CCL investigates patients’ automatic thoughts and cognitions related to anxiety and depression. The scale consists of 26 items and is rated on a 5-point Likert scale. Internal consistency and validity was supported.

#### Executive functioning: n-back task [73]

During the n-back task patients are asked if a letter on the screen matches a letter previously (1-back, 2- back, 3-back) presented for 500 ms with an interval of 2000 ms. First, the patient will be asked to run a test trial, where he will get elaborate feedback about the incorrect responses (‘The previous letter was X, this indicated you had to press the button’). Second, the patient will complete a 1-back trial (two minutes) and a 2-back trial (two parts of 2.5 minutes). Only when the patient performs well on the 2-back (e.g. 2/3 correct responses; a correct response means a correct press or a correct no-press), he will be forwarded to the 3-back part of the task that will also take five minutes (two parts of 2.5 minute). Amount of targets in each condition will be 33 %. Feedback will be given after a correct response (marked by a green V) or a miss (marked by a black X). Working memory load increases as the task progresses from 1- back to 3-back and is suggested to require executive processes. The task will take a maximum of 12.5 minutes. Accuracy of responses (hits – false alarms) and reaction times will be measured. To prevent fatigue of the online assessments the patient will be asked to do the n-back task at another day than the other questionnaires.

#### Treatment preferences [74]

Patients preferences for either CT or IPT will be questioned before treatment by use of a 12-item questionnaire. A similar questionnaire was used in a previous study and adapted for use in the current trial.

### Instruments: biological measurements

Blood samples will be collected at baseline and after six months at the research sites. Blood samples will be collected using EDTA tubes (18 ml), serum tubes (6 ml) and PAX gene tubes (2 ml). EDTA 3 ml tubes will be sent to VUmc where DNA will be isolated followed by analysis of CpG islands adjacent to promoters I and IV. The serum tube, EDTA tubes (6 ml) and PAX tubes will be stored (e.g. in VUmc or hospitals near to the research locations) at −80 °C. After data collection (October 2016) all blood samples will be collected at VUmc. Use of medication and alcohol/drugs use in 24 hours before collecting the blood samples will be registered at baseline and after six months.

### Data-analyses

Data-analyses will include an intention-to-treat analysis and additional subgroup analyses.

### Data-analyses: primary outcome measures

Acute and long-term effects of the interventions (respectively 6 and 12 months) will be analyzed using mixed regression. This will enable us to use all outcome ratings and fit the growth curve of time, enter separate levels for study therapists and clinical sites, and also deal effectively with missing values. Time to recovery and relapse (episode of MDD after remission) and recurrence (episode of MDD after recovery) in the course of follow-up (12 and 24 months) will be analyzed with Cox regression. In addition, we will determine the proportion of patients that show reliable and clinically significant improvement on the outcome measures. Our calculations will be based on the method of Jacobson and Truax [75] which prescribes that Clinical Improvement (CI) is based on both Reliable Change (RC), the extent to which the pre-to-post-difference score is reliable; and on Clinical Significant change (CSC), the extent to which post-treatment scores are clinically meaningful [76]. Although the major question of this study focuses on the intensity of psychotherapy for depression, this study will additionally explore whether the effect of treatment intensity differs between IPT and CBT by testing an interaction between treatment conditions.

### Data-analyses: mechanisms of change

To identify mechanisms of change and the strength of the factors involved, both multilevel models and structural equation models (SEM, using path analysis in Mplus) will be used for mediation analyses. In a recent paper, for example, we used a latent difference score model to analyze mediation and temporality in the context of an RCT [77].

### Data-analyses: biological analyses

Levels of oxytocin, BDNF, methylation profile of the BDNF gene (e.g. CpG I and IV) and RNA marker profiles will be analyzed after finishing data collection. Pre- and post-treatment differences will be computed. The predictive value of the methylation profile of the BDNF gene (e.g. CpG I and IV) and RNA marker profiles on treatment outcome will be investigated.

### Data-analyses: cost-effectiveness

In order to evaluate cost-effectiveness a Cost Effectiveness Analysis (CEA) and Budget Impact Analysis (BIA) will be performed. Within the CEA the difference in societal costs (measured by the TiC-P at baseline and after 3, 6, 9 and 12 months) generated by patients in the two conditions (two sessions versus one session a week) will be related to the difference in clinical effects (measured with the BDI-II and quality- adjusted life-years (QALYS) based on the EQ-5D) over the course of twelve months. Missing cost and effect data will be treated using multiple imputation. Bootstrapping with 5000 replications will be used to estimate 95 % confidence intervals around cost differences and the uncertainty surrounding the ICERs. Uncertainty surrounding the ICERs will be graphically presented on cost-effectiveness planes. Cost- effectiveness acceptability curves [78] will also be estimated. Adjustment for confounders and effect modifiers will be done if necessary. In the budget impact analysis, the effectiveness of interventions will be extrapolated using a simple Markov model over a period of 5 years based on the estimates obtained from the proposed study. Societal, government (Budget Kader Zorg) and insurer perspectives will be considered. Different scenarios will be evaluated including: 1) the intervention is not implemented, 2) the intervention is offered to the whole patient population, 3) the intervention is implemented over a period of 4 years, and 4) the intervention is only offered to subgroups of the potential patient population. These subgroups will be defined based on the results of the study, e.g. subgroups that particularly benefit from the intervention. The total number of patients eligible for the intervention will be estimated based on Dutch incidence and prevalence rates of MDD. Resource utilization is calculated by multiplying the number of eligible patients with the resource utilization rates obtained from the economic evaluation. Different prices will be used to value resource use depending on the perspective of the analysis. Both resource use and annual costs will be presented over a 5-year period for all perspectives. Aggregated and disaggregated total costs per year will be presented for the different perspectives and scenarios. We expect that the largest economic benefits generated by the intervention will be related to reduced productivity losses. A small effect will be expected on the number of sessions needed for sustained recovery. Thus, there will be an increased capacity at mental health institutions to treat depressed patients. This will be taken into account in the BIA as well.

## Discussion

We presented a protocol for studying the session frequency of psychotherapy. Furthermore, we focused on the underlying change mechanisms in psychotherapy and try to capture physiological processes that are hypothesized to be involved during the course of depression and psychotherapy.

The finding that a higher session frequency leads to faster and more improvement of depressive symptoms might lead to adaptions in mental health care organization, where enhancing the session frequency may lead to shorter CBT and IPT treatments and will optimize the (cost) effectiveness of treatments for depression. In this case, not only patients’ suffering, but also the societal burden of depression can be reduced. The finding that a greater session frequency leads to a better and faster treatment outcome might give us insight into the mechanisms of change. Actually, if we find that a greater session frequency leads to better and faster treatment outcomes, we can directly test if specific or non-specific mechanisms of change explain the differences in outcome. Furthermore, by investigating differences in mechanisms of change between the different conditions we might enhance knowledge about the mediating role of nonspecific factors and therapy specific factors in therapy. For example, the finding that only patients who receive CBT improve on the CBT skills will support the hypothesis that learning CBT skills is a therapy-specific mechanism of change. Insight in the mechanisms of change and physiological processes underlying psychotherapy can further enable us to optimize treatments and help us understand human functioning beyond the context of therapy [8].

This study is the first to investigate the hypothesis that, while keeping total number of sessions equal, twice-weekly sessions lead to better outcomes than once-weekly sessions for depression in a head-to- head comparison. Furthermore, by including frequent assessments of hypothesized mechanisms of change, the inclusion of moderators (to specify subsets of patients who respond to specific mechanisms) and using advanced statistical techniques we hope to provide the optimal conditions for detecting mechanisms of change in psychotherapy for depression. Because patients are randomized to type of therapy this study provides the unique opportunity to compare differences between CBT and IPT with regard to session frequency and the mechanisms of change. Another strength of this study is the inclusion of physiological assessments. Over the past decades, there has been an increasing interest in a multidimensional approach for understanding the various forms of mental health disorders. It seems important to not only investigate mental disorders from self-reports or behavioral observations but also to focus on the biological aspects, including genetic and other molecular phenomena [15]. Hopefully, including physiological assessments in the study protocol will contribute to a more comprehensive understanding of depression across the course of psychotherapy.

### Trial status

The trial is in the on-going recruitment phase.

## Abbreviations

ACMTQ: Autonomous and controlled motivation for treatment questionnaire
ASC: Action control scale
BADS: Behavioral activation for depression scale
BDI-II: Beck’s Depression Inventory II
BDNF: Brain-derived neurotrophic factor
BIA: Budget impact analysis
CBT: Cognitive behavioral therapy
CC: Cognition checklist
CCTS-SR: Competencies of cognitive therapy scale-self report
CEA: Cost effectiveness analysis
CI: Clinical improvement
CSC: Clinical significant change
CSPRS: Collaborative study psychotherapy rating scale
CTQ-SF: Childhood trauma questionnaire-short form
EQ-5D(5 L): Quality of life
IPT: Interpersonal psychotherapy
LIFE: Longitudinal interview follow-up evaluation
MDD: Major depressive disorder
MINI-plus: Mini International Neuropsychiatric Interview
PCTS: Performance of CT strategies
QALYS: Quality- adjusted life-years (QALYS)
RC: Reliable change
RDQ: Remission of depression questionnaire
SCID-I: Structural clinical interview for DSM-IV Axis I disorders
SEM: Structural equation models
